# Influence of operational parameters on the fluid-side mass transfer resistance observed in a packed bed bioreactor

**DOI:** 10.1186/s13568-015-0111-x

**Published:** 2015-05-01

**Authors:** Amir Hussain, Martin Kangwa, Ahmed Gad Abo-Elwafa, Marcelo Fernandez-Lahore

**Affiliations:** Downstream Bioprocessing Laboratory, School of Engineering and Science, Jacobs University, Campus Ring 1, 28759 Bremen, Germany; Department of Biotechnology, Faculty of Agriculture, Al-Azhar University, Naser City, Cairo, 11884 Egypt

**Keywords:** Packed bed reactor, External mass transfer, *Saccharomyces cerevisiae*, Alginate, Chitosan, Glucose

## Abstract

The influence of mass transfer on productivity as well as the performance of packed bed bioreactor was determined by varying a number of parameters; flow rate, glucose concentration and polymers (chitosan). *Saccharomyces cerevisiae* cells were immobilized in chitosan and non-chitosan coated alginate beads to demonstrate the effect on external mass transfer by substrate consumption time, lag phase and ethanol production. The results indicate that coating has a significant effect on the lag phase duration, being 30–40 min higher than non-coated beads. After lag phase, no significant change was observed in both types of beads on consumption of glucose with the same flow rate. It was observed that by increasing flow rates; lag phase and glucose consumption time decreased. The reason is due to the reduction of external mass transfer as a result of increase in flow rate as glucose is easily transported to and from the beads surface by diffusion. It is observed that chitosan acts as barrier for transfer of substrate and products, in and out of beads, at initial time of fermentation as it shows longer lag phase for chitosan coated beads than non-coated. Glucose consumption at low flow rate was lower as compared to higher flow rates. The optimum combination of parameters consisting of higher flow rates 30–90 ml/min and between 10 and 20 g/l of glucose was found for maximum production of ethanol.

## Introduction

The increase in fossil fuel usage has resulted in both environmental and health problems due to pollutants produced (Shafiee and Topal 2008). This effect has encouraged researchers in finding alternative, less/non-pollutant cheaper fuel sources like ethanol. Therefore, the use of (bio) ethanol as a fuel has been widely encouraged. The most favored method in ethanol production is through the use of yeast *Saccharomyces cerevisiae* fermentation process in bioreactors (Pscheidt and Glieder 2008; de Jong et al. 2012; Cha et al. 2014; Djordjevic et al. 2014). For many centuries, yeast whole-cells have profoundly been used as a work horse in the production of bioethanol and it is currently the most used microorganism due to its extensively high rate of fermentation of sugars and its high tolerance to by-products produced during fermentation (Matsushika et al. 2009; Hasunuma and Kondo 2012; De Bari et al. 2013; Borovikova et al. 2014). However, as the demand in biofuel increases, there is need in finding both the best bioreactor and fermentation conditions that favor’s higher production and quality. Bioreactors have found their extensive usage in biotechnology and are assimilated in the heart of biotechnological process, being the equipment in which the substrate is effectively bio-converted to the desired products under the microbial cells or enzyme activity (Pilkington et al. 1998; Yu et al. 2007; Crespo et al. 2012; de Jong et al. 2012; Lee et al. 2012; Mathew et al. 2014).

For the past decades researchers have focused both on selecting the best favorable strains for bioconversion as while as in the design of the best bioreactors. To achieve high, effective and economically commercialized industrial production of bioethanol and other bioproducts, there is need to use a bioreactor with immobilized cells and, having an enhanced flow regime that, in turn, will minimize mass transfer limitations. Therefore, the study on the influence of mass transfer on productivity as well as the performance of bioreactor is still needed. These factors are severely affected by both external mass transfer limitations (transfer of reactants to and products from immobilized cell system) and internal mass transfer limitations (rate of transport inside the system (Saini and Vieth 1975; Converti et al. 1985; Anselme and Tedder 1987; Galaction et al. 2011). Cell immobilization technology, the localization of intact cells to a defined region of space with the preservation of catalytic activity presents for the biochemical process industry a radical advance, similar to the introduction of heterogeneous catalysis in the petrochemical and heavy chemical industries (Yu et al. 2007; Willaert and Flickinger 2009; Duarte et al. 2013). This justifies the interest in the research and development advanced materials for biotechnology with the combined effort of scientists from various fields to obtain polymers with well-defined structures and specific chemical, physicochemical, mechanical and biological properties which are used in cell enzyme entrapments (Terada et al. 2006; Duarte et al. 2013). The immobilization technique has found numerous advantages over free cells such as easiness of product separation, reutilization of entrapped cells, maintaining of specific growth, high cells densities and lack of contamination. Additionally, immobilized cells are less susceptible than free cells to the effect of substrate inhibition and pH variations, all these help to improve the overall process. Presently, natural and synthetic polymers such as cellulose, alginate, chitosan, agarose polyurethane, and polyacrylate are being used for cell immobilization with calcium alginate beads being widely used in immobilization of bacteria, yeast, fungi and algae for different bioprocesses (Gòdia et al. 1987; Pacheco et al. 2010; Galaction et al. 2012; Duarte et al. 2013). These polymers have potential application in bioethanol production, vinegar production, and wastewater treatment due to its simplicity, cheap, non-toxic to cells and good mechanical properties. However, there are some disadvantages with their use, such as gel degradation, severe mass transfer limitations, low mechanical strength as it can cause the release of cells from the support and large pore size. To overcome this, a combination of chitosan, a polycationic polymer and alginate, a polyanionic polymer is diffused into the alginate beads to provide a strong ionic interaction between chitosan amino groups and carboxyl groups of alginate which forms a polyelectrolyte complex (PEC) that gives more mechanical support to cells (Yu et al. 2007; Galaction et al. 2012; Duarte et al. 2013).

**Figure 1:**
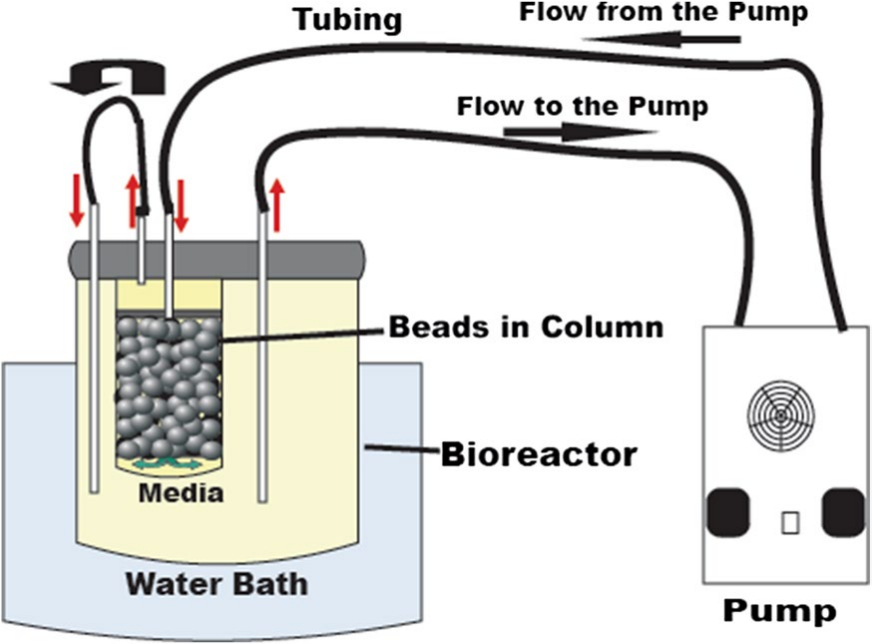
Schematic illustration of a packed bed reactor (PBR) of 100 ml media volume and a 20 ml capacity bead column.

For several decades, traditional setups like membrane, air lift and stirrer tank bioreactors have been used in bioethanol production. However, some drawbacks like, less product yield due to low mass and heat transfer, inefficient conversion of substrate, uneven mixing and shear stress on biocatalysts have been observed. Therefore, there is need in utilizing a reactor that is able to sustain an excellent hydrodynamic regime coupled to reduced overall mass transfer limitations (Saini and Vieth 1975; Pilkington et al. 1998; Karagoz and Ozkan 2014). In this article, we used the packed bed bioreactor (PBR) with one bed containing immobilized beads and a vessel for culture medium, in which the culture medium is circulated from the vessel through the fixed bed and back (Figure 1).

Medium enriched with glucose re-enter the packed bed where it can be re-utilized to convert glucose into ethanol. Toxic metabolites and other by-products are diluted; oxygen and pH can be adjusted to optimal levels. This reactor has several advantages over other bioreactors like, low manufacturing and operating cost, automation process and facility to operate at low temperatures. The preference for fixed bed bioreactor has increased due to its higher sensitivity/effectiveness of immobilized cells or enzymes (Cascaval et al. 2012).

In this article we focused on the operational performance of the immobilized packed-bed bioreactor in the course of physiological and biochemical studies on the substrate uptake of immobilized yeast cells. The reactor was operated in batch mode fermentation; yeast physiology and mass transfer behavior in packed bed reactor were monitored in close relation to parameters such as glucose concentration, medium flow rate and different support materials like alginate beads with and without chitosan coating.

## Materials and methods

### Materials

#### Microorganism

The yeast *S. cerevisiae* (baker yeast) was obtained from DHW Vital Gold, Nürnberg, Germany, while the *S. cerevisiae* Ethanol Red 11 strain was purchased from Fermentis Inc, Marcq-en-Baroeul, France and were stored at 4 and −80°C, respectively.

#### Fermentation medium and cultivation

Minimal media was prepared with 6.7 g/l yeast extract nitrogen base without amino acid, 1.7 g/l ammonium acetate and glucose (2, 4, 10, 20 and 40 g/l) were prepared separately and mixed after sterilizing (121°C, 20 min). Amino acid mixture (100×) was prepared by mixing the following different amino acids; 200 mg l-arginine, 1,000 mg l-aspartic acid, 1,000 mg l-glutamic acid, 300 mg l-lysine, 500 mg l-phenylalanine, 4,000 mg l-serine, 2,000 mg l-threonine, 300 mg l-tyrosine, 1,500 mg l-valine, dissolved in water by adjusting pH 10 with 0.1 N NaOH and used 0.2 µm filter for sterilization. During culturing, 10 ml of amino acids solution was added to a final 1 l media.

Ethanol Red 11 strain was refreshed by streaked onto YPD agar plate (1% yeast extract, 2% peptone and 2% glucose, 2% agar), incubated for 2 days at 35°C. The resulting single colonies were used to start a fresh culture. Twenty milliliters of YPD media (1% yeast extract, 2% peptone and 10% d-glucose) in a 100 ml flask was inoculated with a single colony of Yeast Ethanol Red 11 grown overnight at 35°C with vigorous shaking at 250 rpm. One percent of the pre-culture was used to inoculate 2 l Erlenmeyer baffled flask containing 1,000 ml YPD media final volume. The inoculated flask was incubated on a rotary shaker at 200 rpm and 35°C for 24 h. Furthermore, the cells were collected by centrifugation at 4,000 rpm for 15 min, washed twice with sterile distilled water, centrifuged and re-suspended in sterile water to obtain a dense cell suspension.

#### Calcium alginate beads preparation and yeast immobilization

A sterile sodium alginate solution (2.5% w/v, autoclaved at 121°C, for 15 min, was prepared in 50 mM phosphate buffer at pH 7. For yeast immobilization, 3% final amount of the above obtained cell suspension were mixed with alginate solution. For beads preparation, alginate-yeast solution was drop by drop allowed to dip using 1 ml pipette tip into 200 ml, 180 mM CaCl_2_. Beads were let to harden in this solution for 1 h. Beads were further rinsed three times with sterile 2% NaCl solution and then with sterile water. The alginate beads with diameters between 3 and 4 mm were used in experiments. For the preparation of alginate beads with chitosan coating, the above prepared beads were dipped in sterilized chitosan solution (3% chitosan, 0.1 N HCl, pH 5) for 10 min and later washed 3 times with sterile water.

#### Packed bed reactor and beads packaging

A packed bed bioreactor (100 ml) was purchase from Medorex GmbH, Noerden-Hardenberg, Germany. The bioreactor column has a 2 cm diameter glass vessel for beads package, with one end close and other closed by rubber plug (Figure 1). The reactor was 2/3 filled with beads and temperature was kept at 35°C using a water bath. The immobilized yeast was grown on minimal media with varying factors: glucose (2, 4, 10, 20 and 40 g/l), flow rate (1, 4, 12, 30 and 90 ml/min.) and alginate bead with and without chitosan coating while factors like initial cells amount (3%) and temperature (35°C) were kept constant.

#### Glucose consumption measurements

For immobilized yeast glucose consumption measurements, the DNS method was used. For each measurement, 0.5 ml sample and 0.5 ml DNS solution were mixed in a 1.5 ml Eppendorf tube, vortex for 10 s, and incubated for 10 min at 90°C. After incubation, 40% 0.16 ml potassium sodium tartrate was added, mixed by vortex and placed on ice for 3 min. Two hundred microliter of each sample was measured at 575 nm. The obtained results were compared with calibration curve of different glucose concentration to get actual concentration.

#### Ethanol production measurements

For measurement of ethanol concentration produced in fermentation broth as well as calibration curve preparation, the underlined method was used. Six hundred microliter of fermentation broth samples were collected, transferred to an Eppendorf tube and centrifuged at 9,000 rpm for a min to pellet the cells. Later, 500 µl of the clear supernatant were transferred into a new tube without disturbing the cell pellet, and 5 µl of 1% *n*-butanol was added as an internal standard. The samples were vortexed for 30 s and 1 ml of 25% ethyl acetate was added with a further 5 min vortexing. For phase separation, the samples were centrifuged at 5,000 rpm and the organic phase was used for gas chromatography (GC). For sample measurements, gas chromatograph equipped with flame ionization detector (FID) was used. The columns used were the 30 and 0.25 mm CP-WAX-57CB (Santa Clara, CA, USA). During liquid analysis, temperature programming was employed and the column temperature was initially maintained at 120°C for 2 min and later the oven temperature was increased at a rate of 10°C/min until it reached 150°C. The injector and detector temperature were kept at 150 and 200°C, respectively. The flow rate for carrier gas (helium) was set at 30 ml/min. The injection sample volume was 2 µl. Each set of the experiment and data points were repeated thrice and the reported value was the mean average.

## Results

### Effect of chitosan coating on lag phase and glucose consumption

**Figure 2:**
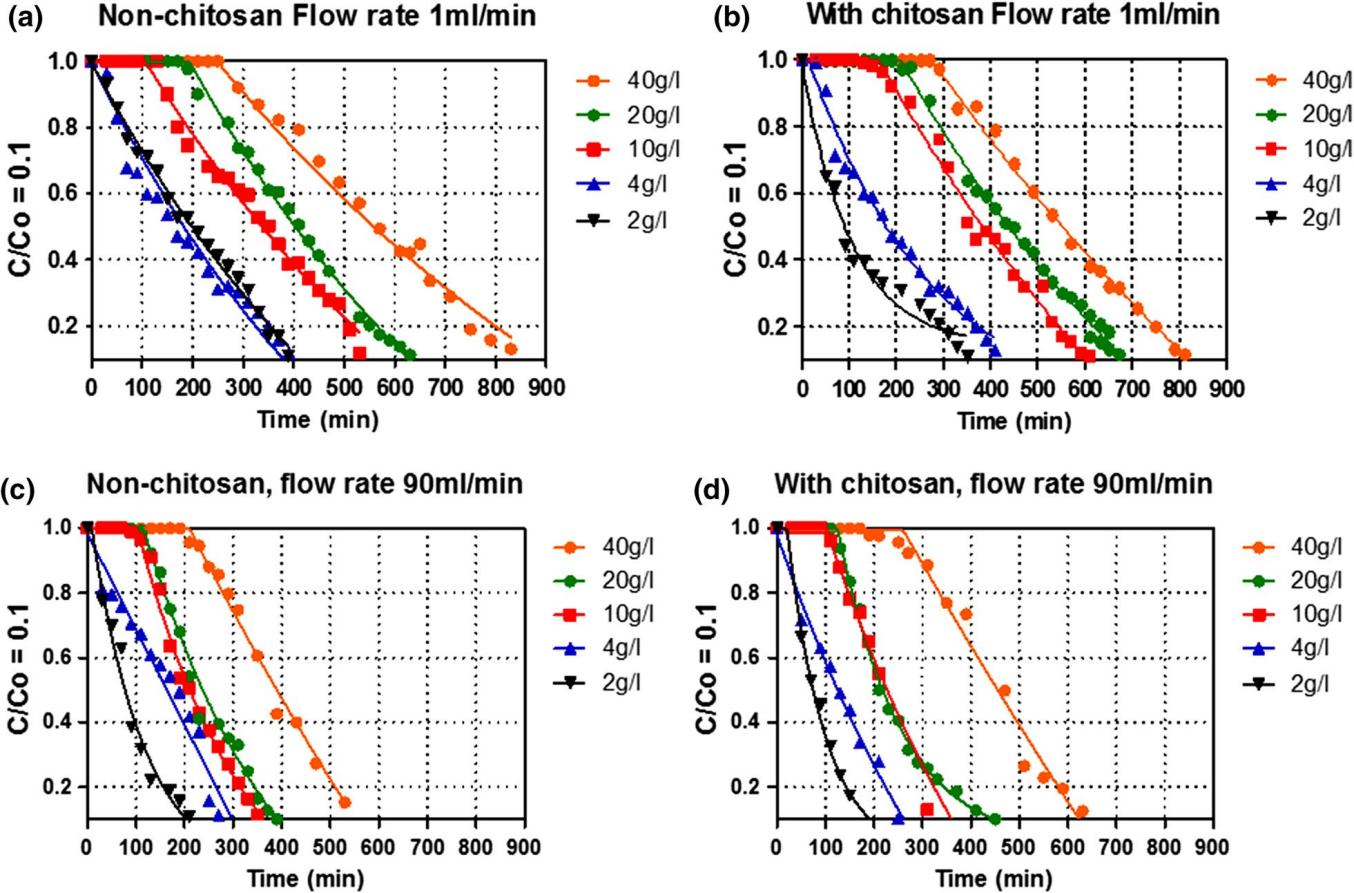
Fermentation profile of immobilized *Saccharomyces cerevisiae* cells in PBR. Effect of flow rate on beads with (**a**, **c**) and non-chitosan (**b**, **d**).

Sequential fermentation experiments with two parameters; flow rate and glucose concentration were varied to understand the effect on lag phase and glucose consumption rate till C/C_0_ of 0.1 in both chitosan and non-chitosan coated calcium alginate beads, where C_0_ represent the initial glucose concentration at time zero, C is the concentration at a particular time and 0.1 (10%) is the remaining glucose in the media. Figure 2a–d shows the flow rates used to determine the effect of chitosan coating on glucose consumption and from the curves we observed two phases: lag and exponential phase. After lag phase, no significant change was observed in both types of beads on glucose consumption with the same flow rate. Additionally, it was also observed that by increasing flow rates; lag phase and glucose consumption time decreased (Figure 2c, d).

### Effect of flow rate and glucose concentration on lag phase

**Figure 3:**
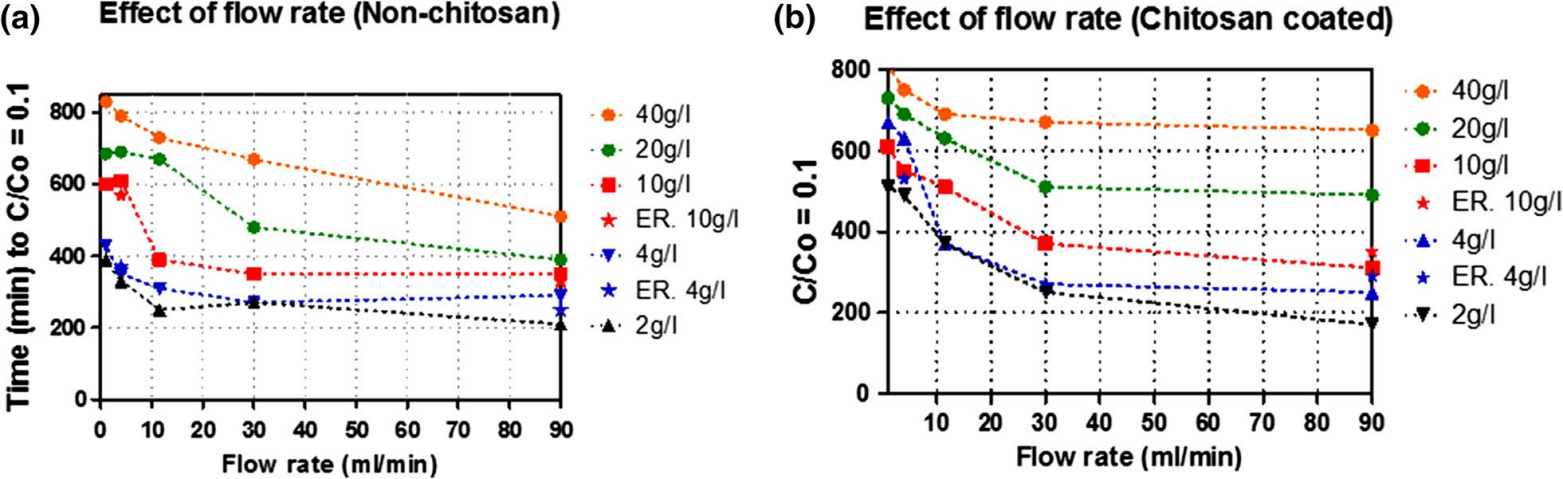
Fermentation profile of immobilized *Saccharomyces cerevisiae* cells in PBR. Effect of flow rate on lag phase using beads with (**a**) and without chitosan (**b**).

The results in Figure 3a, b shows two parameters; flow rate and glucose concentration, varied from 1 to 90 ml/min and 2 to 40 g/l, respectively, having a tremendous effect on lag phase. In the study, it was observed that lag phase of both types of beads decreases by increasing flow rate, moreover longer lag phase was found at higher glucose medium concentration. The maximum time of lag phase was found to be 290 min at lower flow rate of 1 ml/min and 190 min at higher flow rate 90 ml/min when using 40 g/l of glucose. It was also observed that by decreasing glucose concentration from 40 to 10 g/l, lag phase decreased too. Furthermore, no lag phase was found at glucose concentration of 4 and 2 g/l (Figures 2, 3). As shown in Figure 3, non-chitosan coated beads have shorter lag phase as compared to coated beads, indicating an improved mass transfer effect observed at higher flow rate and less inhibition of glucose transfer. While higher flow rate was shown to have a major effect in reducing time on lag phases in both types of beads (Figures 2, 3). To support the above data, fermentation results of the Ethanol Red 11 yeast strain was compared with Baker’s yeast using flow rates of 4 and 90 ml/min and glucose concentration of 4 and 10 g/l. The results show that there is no significant difference in lag phase of two types of yeast.

### Effect of flow rate and glucose concentration on glucose consumption

**Figure 4:**
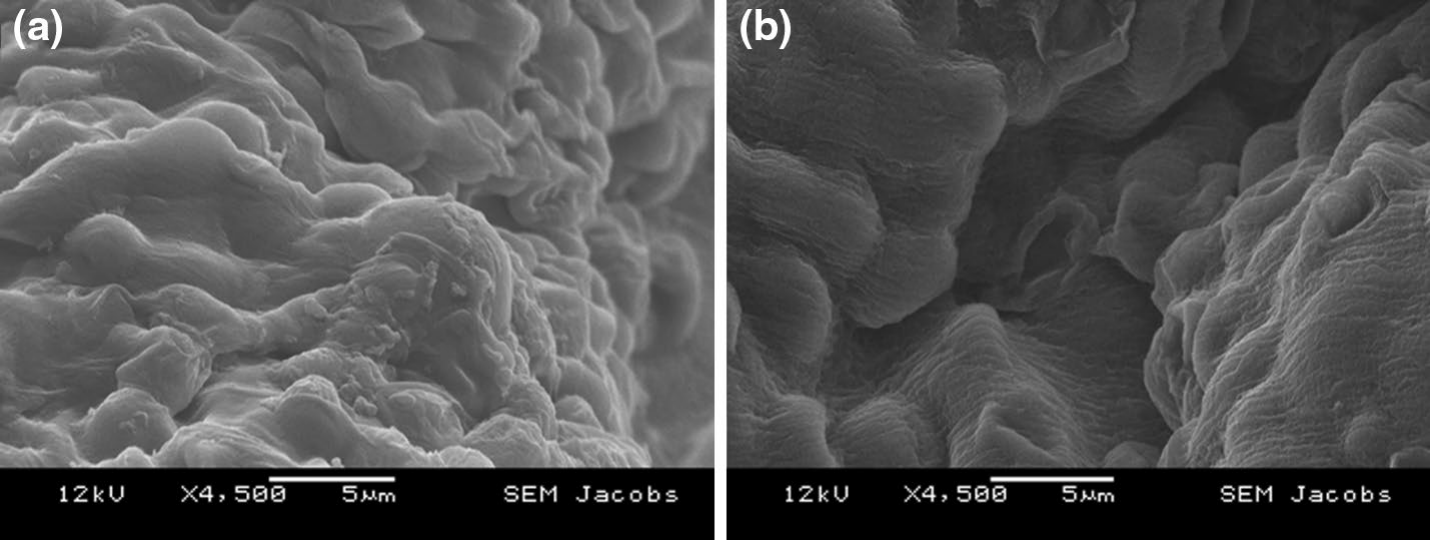
Fermentation profile of immobilized *Saccharomyces cerevisiae* cells in PBR. Effect of flow rate on glucose consumption on beads (**a**) with and, (**b**) without chitosan.

In this study, glucose consumption of up to the level of C/C_0_ = 0.1 was measured so as to understand the performance of the bioreactor and mass transfer properties regarding chitosan and non-chitosan coated beads. Figure 4 shows that by varying the flow rate from 1 to 90 ml/min, time for glucose consumption decreased. The major difference in glucose consumption behavior was observed when using both types of beads at higher flow rate like 90 ml/min. Time for glucose consumption by chitosan coated beads, at 30 and 90 ml/min is rather equal when using higher glucose concentration i.e. 40 and 20 g/l as compared to lower glucose concentration 10, 4 and 2 g/l. Moreover, beads’ having no layer of chitosan, glucose consumption time tends to decrease by increasing flow rate. Further experiments have been performed to compare the *S. cerevisiae* Ethanol Red strain and wild type Baker’s yeast using parameter glucose consumption time. Both strains performance were observed to be relatively equal at 4 and 10 g/l glucose.

### Effect of flow rate and glucose concentration on ethanol productivity and yield

The minimal medium was used in all experiments so that yeast growth rate was at its minimal and cells inside the beads were assumed to be uniform. Experiments were conducted using the above mentioned yeast strains having initial glucose concentrations 4 and 10 g/l and flow rate 4 and 90 ml/min with dilution rate of 0.2 and 4.5 h^−1^, respectively. The effect of flow rate and dilution rate at different glucose concentration on ethanol productivity as well as on ethanol yield is presented in Table 1. It can be observed that when the initial glucose concentration was 4 and 10 g/l, the ethanol productivity increase linearly with the dilution rate from 0.2 to 4.5 h^−1^. An optimal ethanol productivity of 21.9 g/(g h) was obtained when using Ethanol Red strain at D of 4.5 h^−1^ with glucose concentration of 10 g/l. It was also observed that there was no significant difference in ethanol productivity for both *S. cerevisiae* strains at lower flow rate i.e. 4 ml/min, while higher productivity was obtained at higher flow rate (90 ml/min).

**Table 1:** Ethanol productivity and yield by yeast strains

Flow rate (ml/min)	Dilution rate = flow rate/bed volume	Glucose conc. (g/l)	Ethanol productivity		Ethanol yield	
			D × P = (g/g yeast h) at: 300 min		Y(p/s) = Pl − Po/So − Sl at: 300 min	
			B.Yeast	ER.Yeast	B.Yeast	ER.Yeast
4	0.2	4	0.38	0.4	1.11	1.2
4	0.2	10	0.56	0.64	1.0	1.2
90	4.5	4	10.8	12.6	0.63	0.73
90	4.5	10	17.1	19.8	0.48	0.55

*D* dilution rate, *P* product concentration.

## Discussion

In recyclable biocatalyst, the mechanical strength of calcium alginate beads had not fully been found to effectively support entrapped cells. To solve this problem, we focused on using Baker’s yeast immobilized in chitosan coated alginate beads of 4 mm in diameter to facilitate the needed mechanical support. However, the chitosan coating may cause resistance in external mass transfer. The results in Figure 2 indicates that coating has a significant effect on lag phase duration, as it was observed with chitosan coated beads being 30–40 min higher than non-coated beads. The reason is due to the reduction of external mass transfer as a result of increase in flow rate as glucose is easily transported to and from the beads surface by diffusion (Willaert and Flickinger 2009; Galaction et al. 2012; Karagoz and Ozkan 2014).

Our results show an improvement over some literature data, were it was observed that chitosan-covered alginate beads have longer glucose conversion time when compared to alginate beads (Duarte et al. 2013). From the results it can be observed that chitosan acts as barrier for transfer of substrate and products, in and out of beads, at initial time of fermentation as it shows longer lag phase for chitosan coated beads than non-coated. This study gives the significant understanding of both alginate beads with and without chitosan coating as indicated in the differences in lag phases. A number of researchers have been using chitosan coating on alginate beads in order to reduce cell and enzyme release but it has disadvantage on mass transfer and may have an impact on the metabolic activity of cells in beads due to limited substrate supply that ultimately may have an effect on product formation.

Lag phase is considered as the adaptation time of yeast within new environment before the start of fermentation process. The similar effect in Figure 3 was also observed by Irfan et al. (2014) indicating that sugar concentration is critical in fermentation process as it has influence on yeast physiological, growth, rate of production and yield.

The dependence of lag phase on glucose concentration (Figures 2, 3) might be as a result of substrate diffusion and increase in concentration gradient between surface and inner regions of beads (Galaction et al. 2012). The observed prolonged lag phase might be due to higher accumulation of cAMP level stimulated by the effect of glucose on cAMP synthesis as the level of cAMP is higher during initial fermentation time (Ma et al. 1997) i.e. lag phase time and decreased on initiation of exponential growth in yeast cells (Duarte et al. 2013; Djordjevic et al. 2014; Mukherjee et al. 2014).

This summarizes the fact that inter-particle diffusional resistance reduces by increasing velocity around beads (Saini and Vieth 1975; Zhao and Delancey 2000; Galaction et al. 2012). Consequently, chitosan coated beds have more inter-particle diffusional resistance i.e. longer lag phase at early times of fermentation as compare to non-coated at lower flow rate. At this point it can be concluded that lag phase is not due to the physiology of yeast, but it may be due to the resistance in internal diffusion of glucose. This could be due to the fact that lag phase is directly depending on the glucose concentration as well as on flow rate.

**Figure 5:**
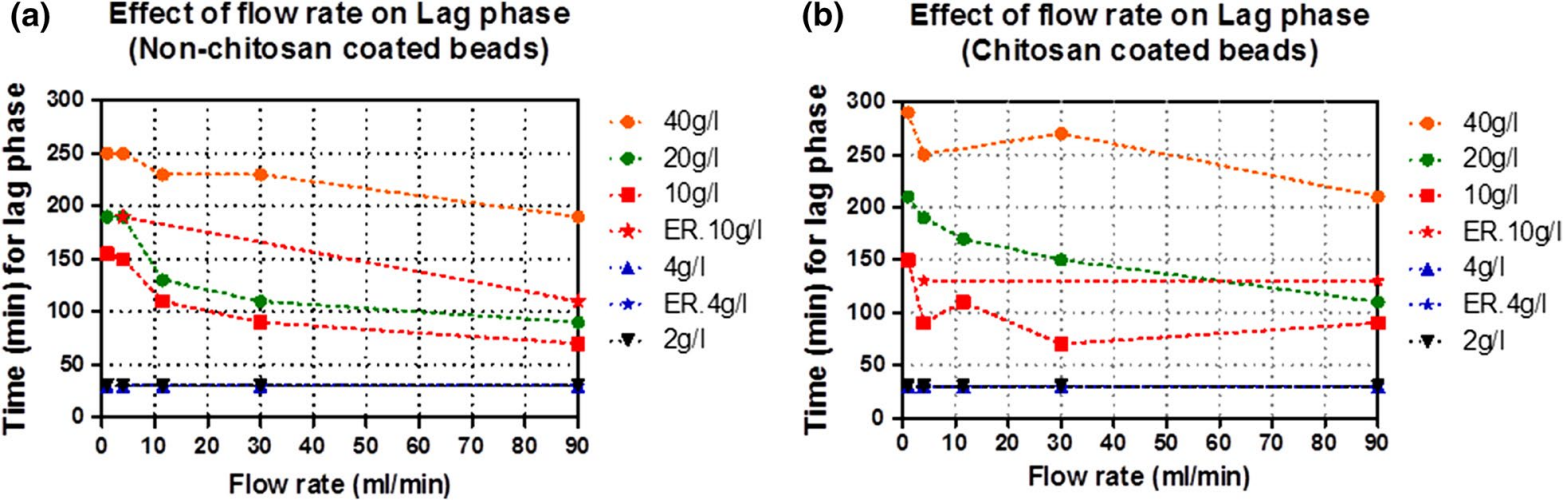
Scanning electron microscopic (SEM) photographs of non-coated alginate beads (**a**), chitosan coated beads (**b**).

Glucose is the most fundamental carbon source playing a central role in metabolic pathways providing energy to living organisms, and for product synthesis. Yeast metabolize glucose via the Embden–Mereyhof Parnas metabolic pathway (Galaction et al. 2012) there-by producing energy necessary for it survival. Furthermore, the efficiency of ethanol production can be affected by glucose concentration and flow rate. From the ethanol production experiments, it was observed that the time for glucose consumption by chitosan coated beads, at 30 and 90 ml/min was rather equal when using higher glucose concentration i.e. 40 and 20 g/l as compared to lower glucose concentration 10, 4 and 2 g/l. This might be due to glucose diffusion resistance that did not reduce even when using higher flow rate. However, this result indicates that chitosan coating characteristics influences glucose internal diffusion at higher glucose concentration. In literature, it was also observed that magnitude of glucose diffusion resistance is directly related to glucose concentration gradient created in and outside of beads (Galaction et al. 2011), indicating substrate inhibition phenomenon, affecting the fermentation performance. In non-chitosan coated beads’ when compared with coated beads, glucose consumption time tends to decrease by increasing flow rate. The reason might be due to the fact that these types of beads did not pose any significant barrier (Figure 5) for glucose diffusion to metabolically active cells. This result was supported by Chen et al. (2012) observation that under scanning electron microscopy (SEM), surface of chitosan-coated beads was rough and compact compared to the non-coated alginate beads, due to strong electrostatic interaction between chitosan and alginate. The interpretation of these results indicates that glucose consumption behavior was not due to the yeast strain, but to mass transfer barrier that might have occurred by layer of chitosan coating on alginate beads and glucose concentration inhibition phenomenon.

As indicated in Table 1, higher ethanol productivity was observed on increasing flow rate and glucose concentration. A higher productivity can be attributed to the improved mass transfer properties when using higher flow rate that might be due to reduced substrate diffusional resistance (Anselme and Tedder 1987; Yu et al. 2007; Matsushika et al. 2009; Pacheco et al. 2010; Bangrak et al. 2011; Mathew et al. 2014). Although higher glucose concentration can give higher productivity, it can also facilitate increase in inter-particle diffusional resistance that enhances the lag phase as shown in Figures 2 and 3. It was also found the enhancement of ethanol production on increasing liquid velocity decrease mass transfer resistance and substrate inhibitory effect (Bangrak et al. 2011).

Duarte et al. (2013) found that the maximum ethanol production during fermentation was after 4 h for non-chitosan coated alginate beads while for coated ones was after 6 h. While it was also reported that hydrodynamics of medium exhibits an important influence on glucose conversion and transfer processes (Cascaval et al. 2012; Galaction et al. 2012; Mathew et al. 2014).

Furthermore, in the case of ethanol yield, the industrial strain Ethanol Red 11 strain has higher yield than Baker’s yeast at all flow rate and glucose concentration. On the other hand at 4 ml/min flow rate and 10 g/l glucose, ethanol yield of both yeast strains was observed to be high as compare to flow rate of 90 ml/min with same glucose. This result is due to higher residence time up to which yield is high (Singh et al. 2009).

Ethanol yield for both strains has been observed to decrease on addition of glucose that might be due to increased substrate diffusional resistance. The magnitude of resistance is directly related to the glucose concentration gradient between the inner and outer regions of beads, consequently concentration gradient can induce substrate inhibition and it was found that there was significant decrease in ethanol yield on addition of sugar concentration in fermentation medium. (Bangrak et al. 2011; Galaction et al. 2011, 2012 Rotaru et al. 2011; Cascaval et al. 2012).

It was also reported that in batch fermentation of *S. cerevisiae*, the ethanol yield was significantly depended on initial glucose concentration and substrate inhibition was notices at high initial glucose concentration (Wendhausen et al. 2001).

Sequential experiments on varying flow rates and glucose in a packed bed bioreactor with immobilized *S. cerevisiae* cells shades significant understanding on mass transfer. Moreover, glucose consumption at low flow rate was lower when compared to when higher flow rates were used. By means of the analysis of the influence of different concentration of glucose and varying flow rate, the optimum combination was found to be that consisting of higher flow rates and between 10 and 20 g/l of glucose. This combination leads to the optimum glucose consumption rate and maximum product formation. The selected system for mixing as well as glucose concentration will be used in the further experiments for internal mass transfer or active pharmaceutical ingredient production in this basket bioreactor.
